# Mutual coupling of neurons in the circadian master clock: What we can learn from fruit flies

**DOI:** 10.1016/j.nbscr.2025.100112

**Published:** 2025-01-17

**Authors:** Charlotte Helfrich-Förster, Nils Reinhard

**Affiliations:** Neurobiology and Genetics, Theodor-Boveri-Institute, Biocenter, University of Würzburg, Am Hubland, 97074, Würzburg, Germany

**Keywords:** *Drosophila melanogaster*, Multi-oscillator system, Clock neurons, Flywire connectome, Neuropeptides, Dual oscillator model

## Abstract

Circadian master clocks in the brain consist of multiple neurons that are organized into populations with different morphology, physiology, and neuromessenger content and presumably different functions. In most animals, these master clocks are distributed bilaterally, located in close proximity to the visual system, and synchronized by the eyes with the light-dark cycles of the environment. In mammals and cockroaches, each of the two master clocks consists of a core region that receives information from the eyes and a shell region from which most of the output projections originate, whereas in flies and several other insects, the master clocks are distributed in lateral and dorsal brain regions. In all cases, morning and evening clock neurons seem to exist, and the communication between them and other populations of clock neurons, as well as the connection across the two brain hemispheres, is a prerequisite for normal rhythmic function. Phenomena such as rhythm splitting, and internal desynchronization are caused by the "decoupling" of the master clocks in the two brain hemispheres or by the decoupling of certain clock neurons within the master clock of one brain hemisphere. Since the master clocks in flies contain relatively few neurons that are well characterized at the individual level, the fly is particularly well suited to study the communication between individual clock neurons. Here, we review the organization of the bilateral master clocks in the fly brain, with a focus on synaptic and paracrine connections between the multiple clock neurons, in comparison with other insects and mammals.

## Highlights

• The fly master clock consists of 240 bilaterally distributed neurons.
• The clock neurons are coupled by multiple synaptic and paracrine connections.
• Some dorsal neurons form strong synaptic contralateral connections.
• Coupling of morning and evening neurons is paracrine and highly plastic.
• Gap junctions & glia cells may contribute to the coupling within the clock network.

## 1 Introduction

Almost all physiological processes in organisms are modulated by the circadian system with a periodicity of 24 h. In animals, bilaterally distributed circadian master clocks in the brain coordinate these 24-h rhythms in the brain and body. Depending on the species, each master clock consists of hundreds to several thousand clock cells that communicate with each other and generate a coherent rhythmic output that is transmitted to downstream neurons and the neuroendocrine system for normal rhythmic functions. In mammals, the master clocks are located in the suprachiasmatic nuclei (SCN) of the hypothalamus just above the optic commissure (Moore-Ede et al., 1984), in mollusks, they are located in basal retinal neurons (Block and Wallace, 1982), and in most insects close to the optic lobes (Helfrich-Förster et al., 1998; Page, 1981a).

### 1.1 Molecular generation of circadian oscillations

Circadian oscillations are generated in individual clock cells by transcriptional/translational feedback loops between clock genes and their protein products (Dunlap, 1999). The core components of the clock are largely conserved in animals: CLOCK (CLK) and CYCLE (CYC) (called BMAL in mammals) are heterodimeric transcriptional activators that drive transcription of the *period* (*per*) and *timeless* (*tim*) genes in flies and the *per* and *cryptochrome* (*m-cry*) genes in mammals. PER and TIM/m-CRY encode repressors that inhibit the function of CLK-CYC/BMAL. Subsequently, PER and TIM/m-CRY are degraded, and the cycle starts again. This happens every ∼24 h. In a second feedback loop, CLK and CYC/BMAL interact with further clock genes, leading to the rhythmic transcription of *Clk* in flies and *Bmal* in mammals. These molecular cycles ultimately lead to rhythmic transcription of clock-controlled genes and, finally, to physiological oscillations in the individual clock cells (e.g. in neurons and glial cells of the brain). In clock neurons, the molecular feedback loops rhythmically control Ca^2+^ levels, membrane resting potential, action potential discharge, and neurotransmitter release (Block and Page, 1978; Flourakis et al., 2015; Harvey et al., 2020; Kuhlman and McMahon, 2004; Paul et al., 2019). In addition, clock neurons show ultradian oscillations in Ca^2+^ and action potential firing that appear linked (coupled) to the circadian oscillations (Liang et al., 2022; Long et al., 2005; Schneider and Stengl, 2007; Stengl and Schneider, 2023). At this point, we will focus on circadian clocks and only briefly address the link between ultradian and circadian rhythms (see chapter 4.2.).

### 1.2 Definition of coupling

The process leading to coherent rhythms of several oscillators is called coupling. Coupling occurs on several levels (Pilorz et al., 2020). (1) molecular coupling coordinates oscillatory gene expression and protein abundance in each rhythmic cell, (2) cellular coupling coordinates the oscillations of different rhythmic cells by signaling molecules and (3) systemic coupling coordinates the rhythms between clocks in different tissues (e.g. the brain and different organs) via autonomous and humoral messengers. Coupling brings the different periods of the oscillators closer together and makes them oscillate in a stable phase relationship. It is not obligatory that they cycle in phase with each other. Coupled oscillators can have different phases, as can be seen when comparing the oscillators in the brain and periphery. The peripheral oscillators usually phase-lag the master oscillators (Kramer, 2009). Also within the brain master clocks, not all clock neurons cycle completely in phase (Evans et al., 2013; Quintero et al., 2003), and the two bilateral master clocks can even be stably coupled in antiphase to each other (see Chapter 1.2.).

In this review, we will focus on the cellular coupling between single clock neurons or certain areas of the master clock as well as on the coupling between the two bilateral master clocks. For a deeper insight into molecular and systemic coupling, the reader is referred to Pilorz et al. (2020).

### 1.3 Coupling between the two bilaterally distributed master clocks

The pioneering work of Terry Page showed that bilaterally distributed master clocks are mutually coupled with each other. He showed this for the mollusk *Bulla gouldiana* (Page and Nalovic, 1992) and the cockroach *Leucophaea maderae*, now renamed *Rhyparobia maderae* (Page, 1982, 1981b; Page et al., 1977). In both cases, the free-running periods of the isolated left and right oscillators within an individual were identical, while the periods of the coupled system were slightly different, being shorter in the cockroach and longer in *Bulla*. This strongly suggests that the two optic lobe pacemakers communicate with each other and that this communication affects the free-running period. Furthermore, following an experimentally generated phase shift in one of the paired oscillators, resynchronization occurred in both animals, but it was slow in *Bulla* and fast in *Rhyparobia*, revealing that the coupling between the two master clocks was weak in *Bulla* and strong in *Rhyparobia*. Weak coupling between the two master clocks was also observed in the beetle *Blabs gigas* (Koehler and Fleissner, 1978) and the cricket *Teleogryllus bimaculatus* (Wiedenmann, 1983). In these animals, differential illumination of the compound eyes (one eye in constant light and the other in constant darkness) was sufficient to dissociate the bilateral master clocks. The clock underlying the constantly illuminated eye lengthened its period, whereas the contralateral clock kept a short period. Wiedenmann (1983) showed, that either the short- or the long-period component disappeared when he removed the optic lobe of the relevant brain hemisphere. Some mammals such as Syrian hamsters appear to have also a weak coupling between the two bilateral SCN because under constant light, the left and right SCN can dissociate into two components that oscillate 180° out of phase (de la Iglesia et al., 2000). Like crickets, one component of the split activity disappeared after the surgical removal of one SCN (Pickard and Turek, 1982). In contrast, a dissociation of the bilateral master clocks in the brain has not been observed in the fruit fly *Drosophila melanogaster* (Helfrich-Förster, 2004). The reason for the different coupling strength between the bilaterally distributed master clocks in different animals may have anatomical reasons. The group of Monika Stengl has traced the master clocks of *Rhyparobia* to the accessory medullae (aMe), small neuropils at the base of the optic lobes that are densely innervated by neurons containing the neuropeptide Pigment-Dispersing Factor (PDF) (Reischig and Stengl, 2003; Wei et al., 2010 and other work of the Stengl group). Both aMe are connected by prominent commissures of PDF fibers in *Rhyparobia* (Reischig et al., 2004; Reischig and Stengl, 2002). The same applies to flies, while no such PDF commissures are present in crickets and beetles (reviewed by Helfrich-Förster et al., 1998). This suggests that the PDF-neurons and perhaps PDF itself are involved in the coupling mechanism (see Chapters 3.2. and 3.4).

### 1.4 Coupling between the neurons within each master clock

Besides the coupling between the master clocks of the two brain hemispheres, a coupling of the individual clock neurons in each master clock is crucial for the generation of a coherent rhythmic output. The SCN are a perfect example to demonstrate this mutual coupling (reviewed in Honma, 2018). Dispersed SCN neurons exhibit circadian rhythms in spontaneous firing with cell-specific periods that have a wide Gaussian distribution ranging between 20 and 30 h and they cycle out of phase with each other (Hastings et al., 2018; Welsh et al., 1995). In contrast, the circadian periods of SCN neurons in a slice culture are synchronized to one another, and the slice-to-slice variability is much smaller than that observed in dissociated cell cultures (Honma et al., 2004; Patton et al., 2016). Free-running periods of behavior were distributed in an even narrower range than in slices (Herzog et al., 1998; Honma et al., 2012). These findings suggest that in an intact SCN, individual neurons communicate to achieve synchronized periodicity representative of the majority of constitutional cellular oscillators. Similarly, mice and fruit flies that possess a wildtype clock only in a subset of the clock neurons show highly variable free-running periods (Frisch et al., 1994; Helfrich-Förster et al., 1998; Low-Zeddies and Takahashi, 2001). These findings suggest that individual clock neurons have periods that differ largely from the species-specific period and underline the importance of intercellular interactions within the master clocks. The individual neuronal oscillations need to be synchronized (coupled) with each other to yield a common period of close to 24 h.

### 1.5 Organization of the master clock in different areas

In addition, the master clocks are organized into different parts. Anatomically, each mammalian SCN is divided into dorsal (shell) and ventral (core) subdivisions (reviewed in Honma, 2018; Mieda, 2020; Pilorz et al., 2020). The SCN core neurons receive projections from the retina, the intergeniculate leaflet, and the raphe nuclei, they respond immediately to environmental light stimuli, have multiple projections to the shell, and convey the light information to the neurons in the shell. The shell neurons have only sparse projections to the core. Instead, most SCN output projections to different parts of the hypothalamus originate in the shell, and the shell neurons show circadian oscillations with higher amplitudes than the core neurons. Core and shell SCN neurons also differ in their neuropeptide composition. The core predominantly consists of vasoactive intestinal polypeptide (VIP) and gastrin-releasing peptide (GRP) expressing neurons, while the shell consists of primarily arginine vasopressin (AVP) neurons with some neurons containing somatostatin, prokineticin 2 and/or cholecystokinin (Mieda, 2023; Onodera et al., 2023). In addition, almost all SCN neurons are GABAergic. The aMe of the cockroach is similarly organized in a core that gets input from the retina and a shell containing mainly output neurons that project to different brain regions, it is also rich in different neuropeptides that are localized in specific regions of the aMe, and GABA is the prevalent neurotransmitter of the clock neurons (Massah et al., 2022; Stengl et al., 2015; Stengl and Arendt, 2016). In flies and several other insects, the circadian clock neurons are dispersed in the lateral and dorsal brain, but like cockroaches, they are rich in neuropeptides and many of them send fibers into the aMe (Beer et al., 2018; Beer and Helfrich-Förster, 2020; Bertolini et al., 2018; Fuchikawa et al., 2017; Kay et al., 2018; Manoli et al., 2023).

### 1.6 Morning and evening oscillators

Finally, there are indications that the master clocks of mammals and insects contain separate morning (M) and evening (E) neurons that control M and E activity and are important for adapting activity rhythms to seasonal changes in photoperiod (Fuchikawa et al., 2017; Gestrich et al., 2018; Helfrich-Förster, 2009, 2014; Pittendrigh and Daan, 1976; Werckenthin et al., 2020; Yoshii et al., 2012). In mammals, M and E neurons have been found in the rostral (anterior) part of each SCN, but there are no specific areas in the rostral SCN that contain only M or only E neurons (Hazlerigg et al., 2005; Inagaki et al., 2007; Jagota et al., 2000). Instead, M and E oscillators form distributed networks in the SCN. In flies, the M oscillators have been localized to a specific group of ventrolateral clock neurons, while the E oscillators appear to be located further dorsal in the dorsolateral brain (Grima et al., 2004; Rieger et al., 2006; Sekiguchi et al., 2024a; Stoleru et al., 2004). Nevertheless, M and E neurons, or at least neurons that affect M and E activity, seem to be also present in the dorsal brain (Rieger et al., 2006), indicating that also flies possess distributed networks of M and E oscillators. Most interestingly, M and E oscillators have a flexible phase relationship to each other, which is necessary to adapt activity to short and long photoperiods (see Jagota et al., 2000 for hamsters, and Menegazzi et al., 2013 for flies). They can also decouple completely from each other and free-run with short and long periods, respectively. This has been shown in flies under constant light conditions (Rieger et al., 2006) or when excess PDF levels are present in the brain (Yoshii et al., 2009).

### 1.7 Purpose of this review

In summary, oscillator coupling in the master clock works on multiple levels, (1) between the bilaterally distributed master clocks, (2) within the different areas of each master clock, and (3) between morning and evening neurons. So far, not all coupling pathways have been revealed on the anatomical and functional level. Since the bilateral master clocks in *D. melanogaster* contain relatively few neurons that are well characterized at the individual level, the fly is particularly well suited to study the connections between individual clock neurons. Here, we review known coupling within the circadian clock network of *D. melanogaster*, possible synaptic and paracrine connections of the different clock neurons, and the resulting possible couplings. In addition, we review findings on the electrical synapses and glial cells and discuss the putative roles in intercellular communication. Whenever possible, we compare the situation in *Drosophila* with that of other animals.

## 2 The circadian clock network in the brain of *D. melanogaster*

*Drosophila's* clock neurons have historically been classified into different groups of Lateral and Dorsal Neurons (LN and DN, respectively) based on their location in the brain, size, anatomy, development, and neuropeptide expression (Ewer et al., 1992; Kaneko and Hall, 2000; Shafer et al., 2006; Helfrich-Förster et al., 2007; Schubert et al., 2018; Reinhard et al., 2022a, 2022b, 2024; reviewed in Dubowy and Sehgal, 2017; Top and Young, 2018; Helfrich-Förster, 2024). The soma size of the clock neurons is quite different and reaches from a maximal diameter of ∼17 μm for the large ventrolateral neurons (l-LN_v_) (Schubert et al., 2018) to a minimum of ∼5 μm for the small centrally projection DN_3_ (s-CPDN_3_) (Reinhard, unpublished). The size of a neuron soma usually correlates with the length of its neurites and the width of its arborizations. Clock neurons that project to the contralateral brain hemisphere and/or show extensive arborizations have larger soma sizes than neurons with short neurites (Schubert et al., 2018). The same was also found for the cockroach *Rhyparobia* (Stengl et al., 2015). In addition, the size of the soma depends on neuropeptide expression. The somata of clock neurons that contain a high amount of a neuropeptide, or several different neuropeptides are larger than those of clock neurons with little or no neuropeptide expression.

**Fig. 1:**
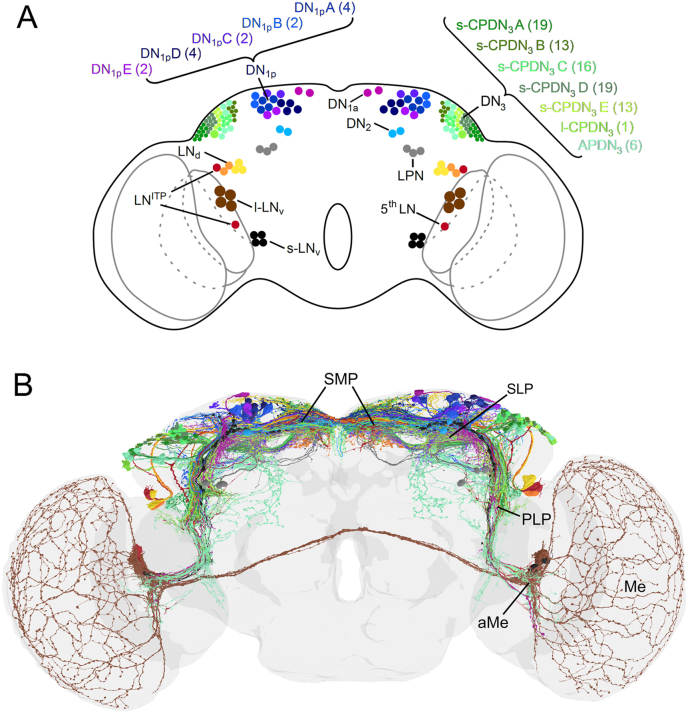
Clock neurons in the brain of *Drosophila melanogaster* **A.** Somata of the clock neurons in the lateral and dorsal brain. The Lateral Neurons (LN) can be divided into the anteriorly located dorsal LN (LN_d_), ventral LN (LN_v_), and 5th LN, as well as the posteriorly located Lateral Posterior Neurons (LPN). Of the six LN_d_, three are Cryptochrome (CRY)-negative (yellow) and three are CRY-positive (orange and red). The LN_d_ in red does express the neuropeptide Ion Transport Peptide (ITP) and is therefore grouped with the ITP-positive 5th LN, in the LN^ITP^. The LN^ITP^ have very similar arborization patterns (see Fig. 2). The LN_v_ can be further subdivided into the large LN_v_ (l-LN_v_) and small LN_v_ (l-LN_v_). Both express the neuropeptide Pigment-Dispersing Factor (PDF). The Dorsal Neurons (DN) can be divided into the anterior DN_1_ (DN_1a_), the posterior DN_1_ (DN_1p_), the DN_2_, and the DN_3_, which include the largest number of clock neurons, of which most have small somata. The DN_3_ are the only clock cluster of which not all somata are drawn. The real number of DN_3_ somata per brain hemisphere can be found in parenthesis behind the different subgroups. The DN_1p_ and the DN_3_ are very heterogeneous in respect of neuropeptide expression and branching patterns. The DN_1p_ can be subdivided into five subgroups (A–E), of which only the first two subgroups (DN_1p_A and DN_1p_B) are CRY-positive. The DN_3_ can even be subdivided into seven subgroups, of which the anterior projecting DN_3_ (APDN_3_) and the large centrally projecting DN_3_ (l-CPDN_3_) have larger somata and are CRY-positive. The other five subgroups project all centrally and have small somata (s-CPDN_3_ A-E). **B.** Reconstruction of all clock neuron arborizations in one female brain stemming from the FlyWire connectome (same color code as in A). The neurites of the clock neurons overlap in four major fiber hubs: the accessory medulla (aMe), a hub in the Posterior Lateral Protocerebrum (PLP), a hub in the Superior Medial Protocerebrum (SMP), and one in the Superior Lateral Protocerebrum (SLP). The l-LN_v_ are the only clock neurons arborizing in the medulla (Me) of the optic lobes. Modified from Reinhard et al. (2024).

While earlier studies counted 150 clock neurons (75 per brain hemisphere) (Shafer et al., 2006), a most recent connectome study found that the largest group of *Drosophila's* clock neurons, the DN_3_, comprises ∼85 instead of ∼40 cells per brain hemisphere (Reinhard et al., 2024). Therefore, the fruit fly possesses ∼240 clock neurons (120 per brain hemisphere) instead of ∼150 (75 per brain hemisphere). The discrepancy between the old and new studies can easily be explained by the small size and dense packing of DN_3_ somata, which makes counting difficult. Fig. 1 shows the distribution of all clock neuron somata and their neurites extracted from the electron microscopic Flywire connectome of a single female *Drosophila* brain (Dorkenwald et al., 2024; Reinhard et al., 2024; Schlegel et al., 2024). From now on, we will always use the number of clock neurons per hemisphere when we refer to cell numbers.

### 2.1 Lateral clock neurons

The Lateral Neurons located in the anterior brain (s-LN_v_, l-LN_v_, 5th LN, and LN_d_) are the so far best-characterized clock neurons, and although they comprise only 15 neurons per brain hemisphere, they are essential for rhythmic behavior under constant darkness (Ewer et al., 1992; Frisch et al., 1994; Helfrich-Förster, 1998). The LN_d_ can be further subdivided into three neurons that express the blue-light sensitive *cryptochrome* (*cry*), the CRY-positive LN_d_, and three neurons that have no or extremely low amounts of CRY (CRY-negative LN_d_). All other LN are CRY-positive. Except for the three CRY-negative LN_d_, all anterior lateral neurons project to the aMe (Schubert et al., 2018; Shafer et al., 2022). In addition to the aMe, there are three other hubs of communication in which many fibers of the LN overlap: one in the Posterior Lateral Protocerebrum (the PLP hub), and respectively one in the superior medial and superior lateral protocerebrum (SMP and SLP hub), a brain area that is often simply called “dorsal brain” (Reinhard et al., 2022b). The three lateral neurons located in the posterior brain (LPN) lie close to the PLP hub and project exclusively to the SMP and SLP (Reinhard et al., 2022a).

### 2.2 Dorsal clock neurons

Although the Dorsal Neurons (DN_1_, DN_2,_ and DN_3_) greatly outnumber the Lateral Neurons, they cannot trigger rhythmic behavior under constant conditions without the involvement of the Lateral Neurons (Sekiguchi et al., 2024b). Therefore, they are often regarded as being lower in the hierarchy of the clock network. But this view is outdated. The Dorsal Neurons crucially contribute to rhythmic behavior under entrained conditions, receive input from different sensory modalities (e.g. from temperature sensing neurons), and connect the clock to diverse output pathways (e.g. sleep centers, neuroendocrine centers, and descending neurons) (Barber et al., 2021; Cavanaugh et al., 2014, 2016; Cavey et al., 2016; Nettnin et al., 2021; Reinhard et al., 2022b, 2024; Saurabh et al., 2024). All 105 DN have arborizations in the SMP and SLP hub, and this is why the “dorsal brain” is extremely rich in clock neuron fibers (Fig. 1B). In addition, several DN arborize in the PLP hub, and few also in the aMe (Fig. 1B). They are therefore closely connected to the LN and undoubtedly play an important role in the circadian system of the fruit fly.

The ∼16 DN_1_ and 87 DN_3_ are an extremely heterogeneous group of clock neurons (Fig. 1A). The DN_1_ can be subdivided into two anterior DN_1_, the DN_1a_, and ∼14 posterior DN_1_, the DN_1p_. The 14 DN_1p_ consist of five further subgroups, four DN_1p_A, two DN_1p_B, two DN_1p_C, four DN_1p_D, and two DN_1p_E, of which the DN_1p_A and DN_1p_B are CRY-positive. The DN_3_ consist of at least seven subgroups. Of these, two subgroups have large somata and show wide arborization patterns (six anterior projecting DN_3_ (APDN_3_) and one large centrally projecting DN_3_ (l-CPDN_3_)) (Reinhard et al., 2024; Sun et al., 2022). The remaining five DN_3_ subgroups consist of numerous centrally projecting neurons with small soma size the fibers of which mostly remain in the ipsilateral brain hemisphere or end in the contralateral brain close to the midline (19 s-CPDN_3_A, 13 s-CPDN_3_B, 16 s-CPDN_3_C, 19 s-CPDN_3_D, and 13 s-CPDN_3_E).

As true for the LN, several DN express CRY. The six CRY-positive DN_1p_ appear to play a particularly important role in M and E activity: The exclusive expression of the clock protein Period (PER) in them is sufficient to ensure almost normal M and E activity, and although these flies lack PER in all other clock neurons, they are not completely arrhythmic under constant conditions, but instead exhibit complex activity rhythms (Sekiguchi et al., 2024). The functions of the CRY-negative DN_1p_ and those of most DN_3_ have not yet been clarified in detail.

## 3 Paracrine and synaptic connections between the clock neurons

The clock neurons communicate with each other through paracrine signaling via neuropeptides and synaptic signaling via classical neurotransmitters. It is important to note that there are important differences between the two communication forms. Neuropeptides are synthesized via ribosomes at the endoplasmic reticulum, modified, and packed into dense core vesicles by the Golgi apparatus, and finally transported to the neurites, where they are stored in dense core vesicles at so-called neuronal varicosities. Upon neuronal excitation, they are released from these varicosities or from other areas of the neuron in a paracrine fashion without requiring classical synapses (Eiden et al., 2022). Once released into the extracellular space, neuropeptides can travel for a certain distance until they reach a neuron that possesses the relevant neuropeptide receptor. Neuropeptide receptors are metabotropic and usually coupled with G-proteins, which initiate different signaling cascades (Hamm, 1998; McCudden et al., 2005; Nürnberg et al., 2024).

In contrast to neuropeptides, classical neurotransmitters are synthesized at the axon terminals, packed in small clear vesicles, and released from the presynaptic neuron into the synaptic cleft upon action potentials (Südhof, 2013). In the postsynaptic neuron, classical neurotransmitters bind to ion channels (ionotropic receptors), which open once activated, or like neuropeptides to metabotropic G-protein receptors, which trigger various cellular responses. In any case, synaptic signaling via ionotropic receptors happens much faster than synaptic signaling via metabotropic receptors and paracrine signaling (Eiden et al., 2022).

Paracrine signaling via neuropeptides does not cause immediate action potentials but rather modulates the activity of the downstream neurons in a long-lasting and state-dependent manner (Eiden et al., 2022). The latter is determined by the sum of inputs from classical neurotransmitters via synaptic transmission. This means that the coupling of two oscillators via classical neurotransmitters and synapses is more direct, faster, and perhaps stronger, but shorter-lived, while the coupling via neuropeptides is plastic and slower but rather long-lasting.

Clock neurons are usually rich in neuropeptides, and this seems to be true for all animals (Helfrich-Förster et al., 1998; Honma, 2018; Mieda, 2020; Stengl and Arendt, 2016). In fruit flies, some clock neurons signal within the network exclusively in a paracrine manner, while most employ additionally fast neurotransmission.

Fly clock neurons that appear to signal within the clock network predominately in a paracrine manner are those that contain the neuropeptide PDF (the l-LN_v_ and the s-LN_v_). While no synaptic connections with other clock neurons have been found for the l-LN_v_ (Reinhard et al., 2024), the s-LN_v_ contain some clear vesicles in their terminals and form very few output synapses (Yasuyama and Meinertzhagen, 2010). PDF receptors are found on many clock neurons (Im et al., 2011; Im and Taghert, 2010), therefore PDF is a main communication signal in the clock network and will be treated in detail in Chapter 3.4. The DN_2_ are the second group of clock neurons that seem to signal mainly via neuropeptides. They appear to express three neuropeptides, Allatostatin C (AstC), short neuropeptide F (sNPF), and Proctolin (Proc), the receptors for which are found on several dorsal and lateral neurons (DN_1p_, DN_3_, LN_d,_ and s-LN_v_), but it remains to be shown whether these neuropeptides serve as communication factors within the clock network or rather as output factors to downstream neurons (Reinhard et al., 2022b, 2024). The LPN express even four neuropeptides (AstA, AstC, Diuretic Hormone 31 (DH31), and DH44 (Chen et al., 2016; Díaz et al., 2019; Ni et al., 2019; Reinhard et al., 2022a, 2024)), but they form also classical synapses (Reinhard et al., 2024). Again, it is not clear, whether AstA, AstC, DH31, and DH44 are communication signals within the clock network or output factors of the clock. However, the receptors are widely expressed within the clock network (Abruzzi et al., 2017; Reinhard et al., 2024).

As do the LPN, the great majority of clock neurons form classical synapses in addition to paracrine signaling. The predominant classical neurotransmitter in the clock network is glutamate. All DNs (except the l-CPDN_3_) are glutamatergic and signal via inhibitory metabotropic glutamate receptors to the LN (Collins et al., 2012, 2014; Hamasaka et al., 2007; Reinhard et al., 2024). Therefore, inhibitory glutamate signaling prevails in the fly clock network as does inhibitory GABA signaling prevail in cockroaches and mammals. Similarly, the s-LN_v_ signal via glycine and inhibitory metabotropic glycine receptors to the DN (Frenkel et al., 2017). Otherwise, most LNs are cholinergic and may signal via excitatory ACh receptors to other LN and DN (Duhart et al., 2020; Reinhard et al., 2024; Schlichting et al., 2019a).

There might be rare cases of clock neurons that signal only via classical synapses (putatively some DN_3_ with very small somata), but this seems to be exceptional and most likely in these cases the relevant neuropeptide has not yet been identified.

### 3.1 Time-dependent remodeling of synaptic connections

In contrast to paracrine signaling, which works over some distance, synaptic signaling depends on cell-to-cell contacts and can vary when the shape of axons is remodeled throughout the 24-h day. Such a remodeling was shown for the s-LN_v_ (Fernández et al., 2008), the LN_d_ (Duhart et al., 2020), and the DN_1a_ (Song et al., 2021). The s-LN_v_ are the best example to demonstrate it. They undergo daily structural remodeling, displaying significantly stronger branching in their dorsomedial projections in the early day than in the early night (Fernández et al., 2008). This rhythm is driven by daily changes in outgrowth and de-fasciculation and changes the synaptic contacts between the s-LN_v_ and the DN_1p_ throughout the day affecting their mutual synaptic crosstalk (Gorostiza et al., 2014). Nevertheless, the abrogation of the daily remodeling did neither disturb paracrine PDF signaling nor locomotor activity rhythms (Fernandez et al., 2020). This suggests that paracrine signaling of the s-LN_v_ is rather independent of daily neuronal remodeling, but that the synaptic coupling between the s-LN_v_ and other clock neurons may vary over time. The same might be true for all clock neurons that use synaptic signaling and has to be taken into account when using connectomes of a single time point to access connectivity as done for the Hemibrain and the Flywire connectome (Scheffer et al., 2020; Schlegel et al., 2024; Zheng et al., 2018).

Despite this limitation, the complete electron microscopy volumes of the fly brain were extremely useful for deciphering the clock network and the synaptic connections within it. In the following, we will review the contralateral and ipsilateral connections between the different clock neurons found by the Hemibrain and Flywire connectome and complement it as far as possible with functional studies.

### 3.2 Contralateral connections

**Fig. 2:**
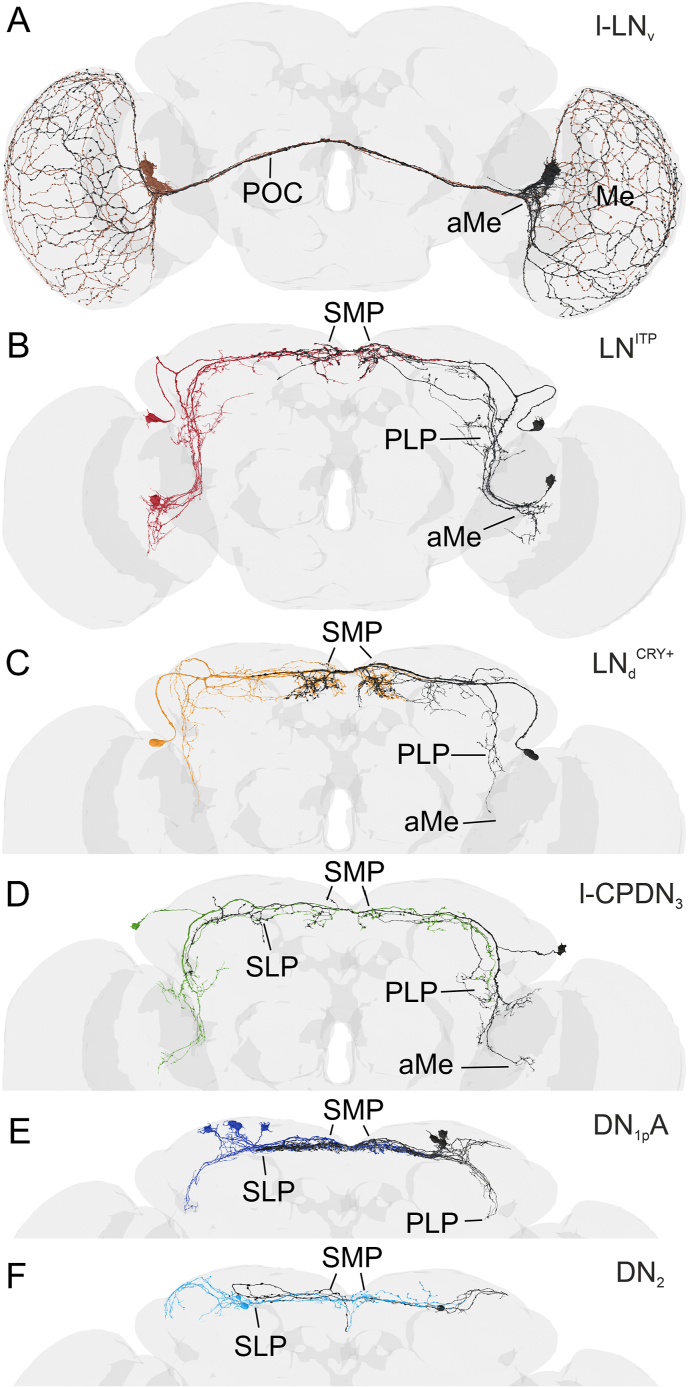
Clock neurons with prominent contralateral projections **A**. The four CRY- and PDF-positive l-LN_v_ project via the posterior optic commissure (POC) into the ipsilateral and contralateral accessory medulla (aMe) and medulla (Me). **B**. The two CRY- and ITP-positive LN^ITP^ project to the ipsilateral and contralateral SMP. Ipsilaterally they arborize in the PLP and aMe. **C**. The two CRY-positive LN_d_ (LN_d_^CRY+^) arborize densely in the ipsilateral and contralateral SMP, and ipsilaterally they send few fibers to PLP and aMe. D. The CRY-positive large centrally projecting DN_3_ (l-CPDN_3_) arborizes in the ipsi- and contralateral SMP, SLP and PLP and sends fibers into the ipsilateral aMe. **E**. The four CRY-positive DN_1p_A have the densest contralateral projections in the SMP and SLP, and ipsilaterally they extend to the PLP. **F**. The fibers of the two CRY-negative DN_2_ remain in the dorsal protocerebrum and invade ipsi- and contralaterally the SMP and SLP. Ipsilaterally they project toward the anterior optic tubercle. On the right hemisphere, one DN_2_ is missing within the FlyWire connectome.

Several clock neurons cross the midline of the brain and project near clock neurons in the contralateral hemisphere. Of 20 identified subtypes, 12 types of clock neurons project contralaterally. However, only six subtypes (l-LN_v_, LN_d_, 5th LN, l-CPDN_3_, DN_1p_A, DN_2_) show extensive branching in the contralateral hemisphere (Fig. 2) (Reinhard et al. 2022a, 2024, 2022b, Schubert et al., 2018). Of the other six subtypes, either only a few fibers reach the contralateral side, or they show dense contralateral branching very close to the midline.

The widest contralateral arborizations are formed by prominent PDF-positive fibers that connect both aMe and project further into the distal layer of the medulla (Helfrich-Förster et al., 2007; Schubert et al., 2018). These stem from the l-LN_v_ and, very similar to cockroaches, they run in the posterior optic commissure (POC) to the contralateral optic lobe (Fig. 1, Fig. 2A). The neuropeptide PDF stemming from the l-LN_v_ was hypothesized to be released from varicosities into the contralateral aMe and to signal to other clock neurons arborizing there (Helfrich-Förster et al., 2007). Indeed, many of the clock neurons that arborize in the aMe express PDF receptors (DN_1a_, LN_d_, 5th-LN, s-LN_v_, and putatively the l-CPDN_3_ and APDN_3_) (Im and Taghert, 2010; Shafer et al., 2008) and PDF was shown to be an important synchronizer of the oscillations of several clock neurons (Liang et al., 2017; Lin et al., 2004; Peng et al., 2003; Yoshii et al., 2009) (see also Chapter 3.4.). The l-LN_v_ are unique clock neurons because they are the only ones that arborize extensively in the optic lobes (Fig. 2A) and might have a special role in the light-input pathway of the clock (Au et al., 2023; Parisky et al., 2008; Shang et al., 2008; Sheeba et al., 2008). Most interestingly, they maintain strong molecular oscillations only under light-dark cycles and stop oscillating under constant darkness (Helfrich-Förster et al., 2001; Lin et al., 2004; Peng et al., 2003; Shafer et al., 2002; Veleri et al., 2003; Yang and Sehgal, 2001). Very similarly, VIP neurons in the core of the SCN that receive light input from the retinohypothalamic tract have strong oscillations under light-dark cycles and weak ones under constant darkness (Antle and Silver, 2005). VIP and PDF appear to fulfill similar roles in the circadian system of mammals and flies (see Chapter 3.4.).

All other contralateral connections between the clock neurons run via the dorsomedial commissure and do not reach the contralateral aMe (Fig. 2B–F). The contralaterally running fibers of the three CRY-positive LN_d_ and 5th LN reach the contralateral *pars lateralis*, terminate close to the DN_1p,_ and show strong branching in the SMP and PLP-hub (Fig. 2B–C). These neurons belong to the E oscillators (Yao and Shafer, 2014). The arborizations of one LN_d_ and the 5th LN are very similar, and both express the Ion Transport Peptide (ITP), which is why they are grouped as ITP-positive LN (LN^ITP^) (Fig. 1, Fig. 2B) (Schubert et al., 2018). Besides PDF, ITP could serve as a second potent bilateral coupling factor in the clock network, and indeed a recent study has identified ITP receptors in several DN (Gera et al., 2024). ITP appears to have a functional counterpart in the mammalian SCN: the neuropeptide arginine-vasopressin (AVP). AVP is expressed in the dorsal parts of the SCN and has been identified, in addition to VIP, as a coupling factor between individual clock neurons (Mieda, 2019; Mieda et al., 2015). Like VIP, AVP also signals within the ipsilateral SCN of mammals, and the same seems to be true for ITP (Gera et al., 2024). In addition to paracrine signaling, the LN^ITP^ form classical synapses across hemispheres (Reinhard et al., 2024).

The next conspicuous contralaterally projecting clock neurons are the large centrally projecting DN_3_ (l-CPDN_3_) (Fig. 2D). The fibers of the l-CPDN_3_ reach contralaterally down to the PLP hub and form synaptic connections with the contralateral small centrally projecting DN_3_D and E (s-CPDN_3_D and s-CPDN_3_E). The l-CPDN_3_ are the only DN that use acetylcholine instead of glutamate as a neurotransmitter (Reinhard et al., 2024) and from their morphological appearance, they look more similar to the LN^ITP^ than to any other DN. Further studies must show whether they play a similar role as E neurons as do the LN^ITP^.

The DN_1p_A are less conspicuous than the so far mentioned clock neurons, but they are the ones that form by far the strongest contralateral synaptic connections within the clock network (Fig. 2E) (Reinhard et al., 2024). They target the contralateral s-CPDN_3_C and D and the LN^ITP^. The latter are in turn contacting the ipsilateral and contralateral DN_1p_A, and s-CPDN_3_ (Reinhard et al., 2022b, 2024). This makes the DN_1p_A an unparalleled strong candidate for coupling clock neurons (Reinhard et al., 2024).

The last DN with contralateral projections are the DN_2_ (Fig. 2F). Besides their central role in regulation of temperature preference (TRP) (Kaneko et al., 2012) not much is known about their function. If this is the only function of the DN_2_ within the circadian network remains to be seen.

**Fig. 3:**
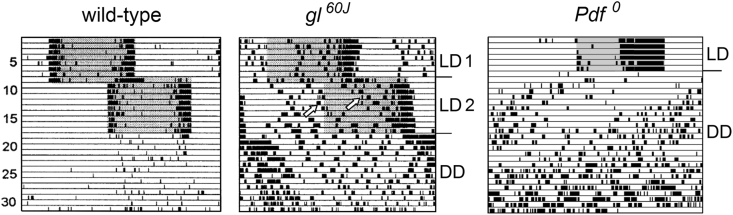
Typical actograms of a wildtype fly and two mutants (*gl*^*60J*^ and *Pdf*^*0*^) that putatively affect the coupling between the clock neurons All flies were first recorded under 12:12 h light-dark cycles (LD, see shaded areas for the light phase). After 8 days the light-dark cycle (LD1) was phase-delayed by 8 h (LD2) for the wild-type fly and *glass* mutant (*gl*^*60J*^) to show that they can re-entrain quite fast to a new light-regime. In contrast to the wild-type fly, the *gl*^*60J*^ mutant showed a broad scattered activity of which some activity components re-entrained only slowly to the new light regime (open arrows). After transfer to constant darkness (DD), the broad and scattered activity of the *gl*^*60J*^ mutant persisted, but the fly did not become arrhythmic. In contrast, the *Pdf*^*0*^ mutant, which was transferred to DD directly after the first LD cycle, initially showed a narrow activity band band that continuously broadened leading finally to arrhythmicity. This mutant free-ran with a short period before it became arrhythmic. Modified from Helfrich-Förster et al. (2001).

In summary, the two bilateral master clocks of the fruit fly are strongly coupled to each other via multiple paracrine and synaptic pathways. This makes it unlikely that the oscillations of the two master clocks can easily decouple from each other, and indeed such a decoupling has not been observed experimentally in wild-type flies. As stated above, the synaptic coupling of two oscillators via classical neurotransmitters is more direct, faster, and perhaps stronger than the coupling via neuropeptides. Consistent with this, *glass* mutants flies (*gl*^*60J*^) lacking the DN_1p_ and consequently also the strong synaptic coupling via the DN_1p_A show weak and unusually scattered activity rhythms (Fig. 3). Since *gl*^*60J*^ mutants lack all DN_1p_, future studies will need to determine whether ablation of only DN_1p_A is responsible for the scattered activity rhythms. It is also not clear whether the changed behaviour is due to a decoupling of the bilateral master clocks. The broad band of activity exhibited by the flies under constant conditions suggests a broad but stable phase relationship between the bilateral master clocks. While *Pdf*^*0*^ mutants, which lack the long-lasting modulatory effects of PDF and become arrhythmic after several days in constant darkness (Renn et al., 1999), flies without DN_1p_ remain rhythmic under such conditions (Fig. 3). *Pdf*^*0*^ mutants initially do not show a broad activity band, suggesting that the coupling of the bilateral master clocks is maintained even in absence of PDF. However, the coupling between individual clock neurons appears weaker leading to a continuously broadening activity that finally results in arrhythmicity (see Chapter 3.4.).

### 3.3 Ipsilateral connections - M and E neurons

**Fig. 4:**
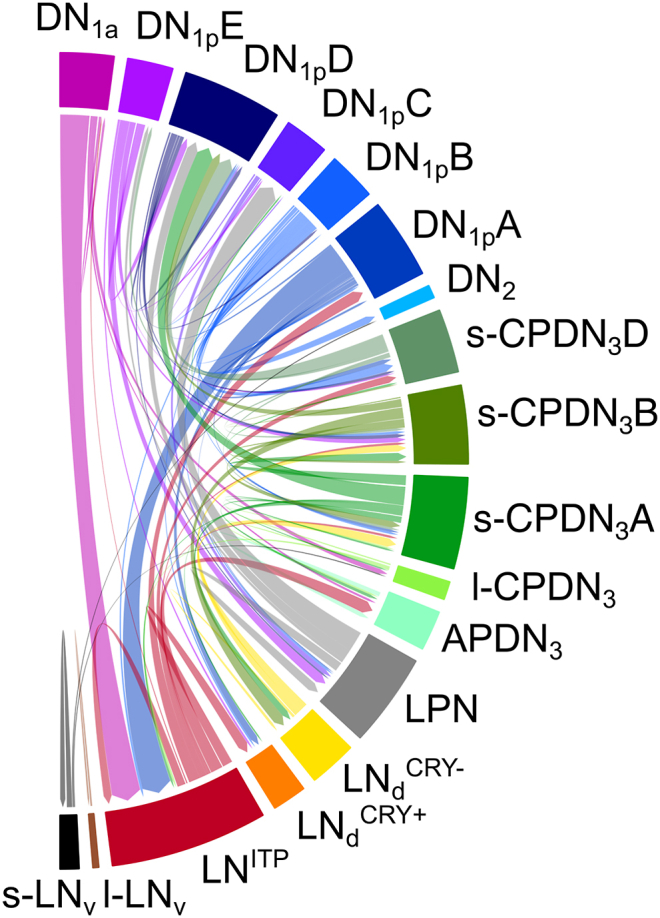
Ipsilateral synaptic connections between the clock neurons according to the hemibrain. The size of the colored bars at the margin of the half-circle, which represents the different clock neurons, is plotted proportionally to their synaptic connections, meaning that those clock neurons that have virtually no synaptic connections within the ipsilateral clock network, such as the four s-LN_v_ and four l-LN_v_ are represented by very slim black bars, whereas the ones that have plenty connections such as the two LN^ITP^ are represented by thick dark red bars. The direction of the arrow indicates the flow of information. Only connections with >9 synapses were taken into account. Please note that the l-LN_v_ synapse only onto each other, and that the s-LN_v_ have extremely few synapses with other clock neurons, but not with the E neurons (LN^ITP^ and LNd^CRY+^). See text for further explanations. Modified after Reinhard et al. (2024).

As already shown for the contralateral connections, the clock neurons within each master clock also communicate with each other. The multiple synaptic connections can be well visualized from data of the hemibrain (Scheffer et al., 2020), which lacks by its nature the contralateral connections (Fig. 4).

Most groups of DN (violet, blue, and green colors) are very well connected with other clock neurons of the ipsilateral master clock, especially the DN_1_ and s-CPDN_3_ but also the LPN (grey) and the E neurons (LN^ITP^ and LN_d_^CRY+^). The LN^ITP^ E neurons receive particularly strong input from the DN_1a_ and the DN_1p_A, while they signal back to the DN_1p_A and in addition to two groups of DN_3_, but not to the DN_1a_ (Fig. 4) (Shafer et al., 2022). From their neurotransmitter content, we know that the input to the E neurons is inhibitory glutamatergic, whereas the E neurons transfer activating synaptic input via acetylcholine (Reinhard et al., 2024). This indicates that the activity of the E neurons and consequently locomotor activity in the afternoon and evening is inhibited by the DN_1p_A and the DN_1a_. Indeed, such an inhibitory effect of the DN_1p_A on activity in the afternoon has been demonstrated experimentally. When a subset of DN_1p_ including the DN_1p_A is optogenetically activated, they inhibit activity in the afternoon and thus prolong the siesta of the flies (Guo et al., 2016). Therefore, the DN_1p_ have been also named “siesta neurons”. Similarly, the DN_1a_ inhibit the evening neurons in particular under warm temperatures (Alpert et al., 2020; Li et al., 2024).

The DN_1p_A and DN_1a_ modulate also morning activity, but not via synaptic connections. The DN_1p_A affect the M neurons indirectly via CNMa (Mao et al., 2024) and the DN_1a_ signal via CCHamide1 (CCHa1) to the M neurons (Fujiwara et al., 2018; Kuwano et al., 2023). The interaction between the s-LN_v_ with the two DN_1a_ is reciprocal. The DN_1a_ have PDF receptors while CCHamide receptors are present on the s-LN_v_. Thus, the DN_1a_ and s-LN_v_ are coupled via CCHa1 and PDF, and this interaction fine-tunes the timing and level of morning activity (Fujiwara et al., 2018). In addition, the coupling of the DN_1a_ and the LN_v_ is important for the startle response to light-on in the morning (Song et al., 2021). CCHamide and PDF work both modulatory excitatory, an effect that is balanced by inhibitory actions of the two additional neurotransmitters of the s-LN_v_ and DN_1a_ (glycine and glutamate, respectively) (Collins et al., 2014; Frenkel et al., 2017; Hamasaka et al., 2007). The lack of synapses between the s-LN_v_ and the DN_1a_ in the Hemibrain and FlyWire connectome might be due to the daily remodeling of these neurons (Fernández et al., 2008; Song et al., 2021). In any case, the synaptic and paracrine coupling of the DN_1a_ and s-LN_v_ appears suited to adapt morning activity to different environmental conditions.

Most interestingly, there exist no reciprocal synaptic connections between the M (s-LN_v_) and E neurons (LN^ITP^ and LN_d_^CRY+^) (Fig. 4) (Reinhard et al., 2024) and there are also no other reports on a synaptic coupling of these neurons. The communication between M and E neurons is exclusively paracrine and works mainly via PDF. This allows a plastic coupling between M and E neurons, which is necessary for an adequate adaptation to seasonal changes in day length. M and E activity couple to dawn and dusk respectively, meaning that M and E activity have a large phase relationship under long summer days. PDF signaling is increased under long days (Hidalgo et al., 2023) and slows down (=delays) the oscillations of the E neurons leading to a late E activity (Liang et al., 2016, 2017; Menegazzi et al., 2017; Schlichting et al., 2019b; Vaze and Helfrich-Förster, 2021). How the oscillations of the M neurons are accelerated (= phase advanced) is less clear, but it was shown that the s-LN_v_ show only phase advances in response to thermogenetic activation (Eck et al., 2016) and that PDF receptor activation in the s-LN_v_ advances morning activity (Choi et al., 2012). In addition, the DN_1a_ that express CRY might be activated by light in the morning and then activate the s-LN_v_ via CCHa1, which might lead to a phase advance. So far, the interaction of DN_1a_ and s-LN_v_ has not been investigated under long days, but future experiments can test this hypothesis.

**Fig. 5:**
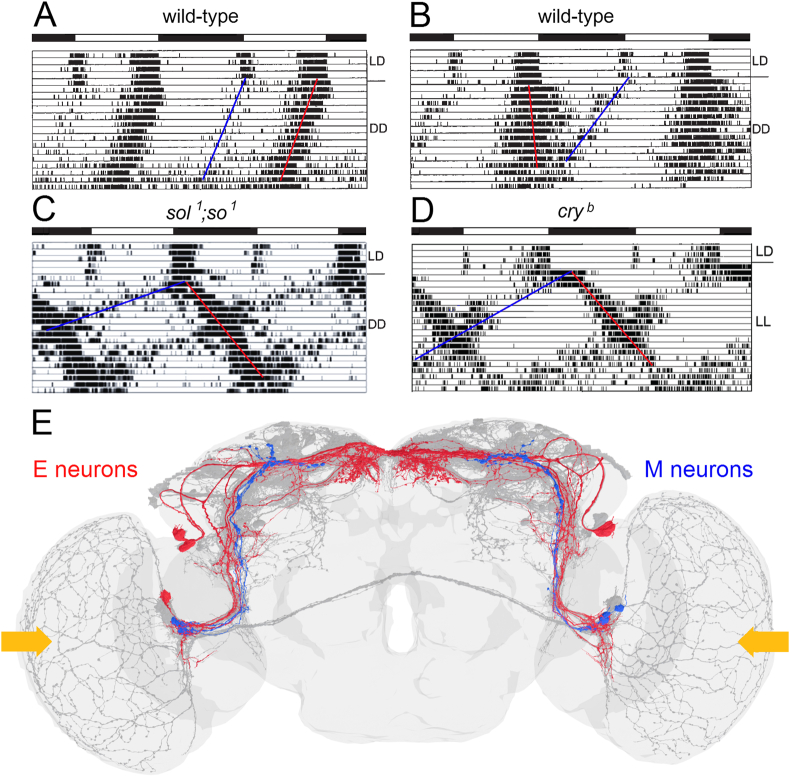
Morning (M) and evening (E) activity of wild-type and mutant flies, which are controlled by M and E neurons **A**. Double-plotted actogram of a wild-type fly, in which the M activity (blue line) persists in constant darkness (DD) and free-runs in parallel to the E activity (red line). **B**. Double-plotted actogram of a wild-type fly, in which the M activity free-ran with a shorter period than the E activity. **C.** Typical activity pattern of a *sol*^*1*^*;so*^*1*^ mutant that has extraordinarily high PDF levels in the central brain, which shortens the free-running period of the M oscillator and lengthens that of the E oscillator leading to an internal desynchronization of both oscillators. **D.** Typical activity pattern of a *cry*^*b*^ mutant in LD and in constant light (LL). Due to not functional CRY, the fly remains rhythmic in LL, and light from the compound eyes to the l-LN_v_ increases PDF secretion into the accessory medulla, shortening/lengthening the periods of M and E oscillators, respectively, and causing internal desynchronization. E. M (blue) and E (red) clock neurons in the *Drosophila* brain. The yellow arrows indicate the light input to the l-LN_v_ which activates them and leads to enhanced PDF secretion into the accessory medulla. Modified from Helfrich-Förster (2024).

The plastic coupling of M and E neurons via paracrine signals can also explain the internal desynchronization between M and E neurons happening in mutants that have extraordinarily high PDF levels in the central brain (Fig. 5C) (Yoshii et al., 2009). In such flies, the M oscillator is constantly accelerated and the E oscillator is constantly slowed down. Consequently, the M oscillator free-runs with a short period and the E oscillator with a long period (Fig. 5C). The same happens under constant light conditions since light activates the PDF neurons leading to enhanced PDF secretion (Fig. 5D and E) (Rieger et al., 2006).

The cellular basis of PDF signaling.

The neuropeptide PDF appears to be the most important signaling factor between the clock neurons within the clock network of insects, and it is so far the only factor for which the cellular mechanisms of coupling have been investigated. Therefore, a special paragraph is devoted to PDF. For a comprehensive review of PDF signaling and its role in the circadian clock of insects, the reader is referred to Shafer and Yao (2014).

As mentioned, PDF is present in the s-LN_v_ and l-LN_v_ of *Drosophila*, which are clock neurons with very different morphology and function (Helfrich-Förster, 1995, 1997; Helfrich-Förster et al., 2007). The l-LN_v_ connect the two bilateral aMe and play a role in the light input pathway to the circadian clock network, which becomes most evident under long photoperiods. They oscillate with high amplitude under light-dark cycles, but not under constant darkness, and appear therefore not to be self-sustained autonomous circadian oscillators. In contrast, the s-LN_v_ play a main role in maintaining circadian rhythmicity under constant darkness. *Pdf*^*0*^ mutants become arrhythmic after some time in constant darkness as can be seen in Fig. 3, and this effect is due to the absence of PDF in the s-LN_v_ (Grima et al., 2004; Renn et al., 1999). At the same time, the s-LN_v_ are M neurons (Grima et al., 2004; Rieger et al., 2006; Stoleru et al., 2004) and *Pdf*^*0*^ mutants have weak M activity (Fig. 3). Despite the different roles of the s-LN_v_ and l-LN_v_, both types of neurons interact with each other and their effects on the fly circadian system are not easy to separate (Shafer and Taghert, 2009). The l-LN_v_ release PDF in a paracrine manner mainly into the aMe (and into the medulla), while the s-LN_v_ do so mainly into the dorsal protocerebrum (the SLP and SMP). In both regions, PDF affects the clock neurons that possess the PDF receptor. Among these are the s-LN_v_ themselves, the LN^ITP^, the CRY-positive LN_d_, the DN_1a_, the DN_1p_A and B, and a few DN_3_ including the l-CPDN_3_.

The PDF receptor is G-protein coupled and its activation leads to an increase in cAMP in most *Drosophila* clock neurons (Lear et al., 2009; Mertens et al., 2005; Shafer et al., 2008) and affects Ca^2+^ levels in some (Mertens et al., 2005; Seluzicki et al., 2014). The same is true for the cockroach, *Rhyparobia maderae* (Gestrich et al., 2018; Wei et al., 2014). As important second messengers cAMP and Ca^2+^ can have several effects in the downstream neurons (Pollard et al., 2017). Increases in cAMP levels can activate hyperpolarization-activated cyclic nucleotide-gate (HCN) channels, stimulate the protein kinase A (PKA), activate the cyclic-AMP response element binding protein B (CREB2), and lead to changes in transcription. Ca^2+^ can depolarize the neurons, increase their firing frequency and neurotransmitter release, but also bind and activate many proteins. Some of these effects have been shown for the circadian clock of *Drosophila*. For example, the *Drosophila* HCN channel, I_h_, is expressed in the s-LN_v_ and necessary to achieve the high-frequency bursting firing pattern characteristic for these neurons (Fernandez-Chiappe et al., 2021). Thus, I_h_ is fundamental for the timed release of PDF-filled dense core vesicles at the s-LN_v_ terminals and hence for the temporal coordination between individual s-LN_v_ and circadian behaviors. *Drosophila* CREB2 has been shown to interact with the core molecular clock feedback loop (Belvin et al., 1999). Furthermore, PKA signaling enhances the stability of the Period (PER) and Timeless (TIM) proteins, resulting in delays in the phase and slowing-down of the circadian clock period within target clock neurons, e. g. the E neurons (Li et al., 2014; Seluzicki et al., 2014).

Most likely due to different PDF receptor signalosome compositions, the effects of PDF were different in the different clock neurons of *Drosophila* (Duvall and Taghert, 2013). Some clock neuron classes rely on PDF for the maintenance of coherent and synchronized molecular oscillations, including the PDF-expressing s-LN_v_ themselves and the DN_1p_ that as a sum become quickly arrhythmic without PDF (Lin et al., 2004; Yoshii et al., 2009). This PDF action can be realized via the HCN channels. The fact that molecular rhythms within the s-LN_v_ become progressively desynchronized in *Pdf*^*01*^ mutants under DD conditions fits well with the observation that locomotor rhythms of *Pdf*^*01*^ mutants weaken progressively over the subsequent circadian cycles in constant darkness (Fig. 3). However, other clock neuron classes, such as the LN_d_ E neurons display increased synchrony in the absence of PDF, and cycle with high amplitude and shortened period (Yoshii et al., 2009). In these neurons, PDF clearly has period-lengthening effects (most likely via PKA signaling), and this fits the short free-running periods *Pdf*^*0*^ mutants display in their locomotor activity rhythms before they become arrhythmic (Fig. 3).

In summary, PDF has different effects on the various PDF receptor-expressing neurons, but on average it appears to be a synchronizing factor with period-extending effects. This is reminiscent of the actions of VIP in the SCN of mammals (Aton et al., 2005; Harmar et al., 2002; Hastings et al., 2014). Like PDF receptors, VIP receptors, VPAC2, are expressed in many clock neurons in the dorsal regions of the master clocks, and mice lacking VIP or VPAC2 show weak circadian rhythms with short periods. Paracrine VIP signaling is sufficient to restore cellular synchrony and amplitude of SCN clock neurons in mice lacking VIP (Maywood et al., 2011). Even more interestingly, PDF and VPAC2 receptors are homologous G-protein coupled receptors, the activation of which leads to an increase in cAMP levels. Increasing cAMP in the SCN of mice that lack VPAC2 receptors can temporarily restore cellular synchrony in the SCN (Atkinson et al., 2011). Furthermore, optogenetic manipulation of intracellular cAMP levels in the SCN shifts molecular and behavioral circadian rhythms, demonstrating that intracellular cAMP is a key molecule in the organization of the circadian neural network of the SCN (Ono et al., 2023). cAMP may play a similar role in the circadian neuronal network of *Drosophila*.

There is another interesting parallel between PDF and VIP in flies and mice: PDF and VIP appear to exert a phase-delaying influence on the oscillations of at least some of the clock neurons that express the PDF or VPAC2 receptors, respectively. In mice, the oscillations of the molecular feedback loop, the resting potential, and Ca^2+^ have a later phase in the sum of VPAC2 receptor-expressing clock neurons than in the sum of VIP neurons (Patton et al., 2020). As mentioned earlier, PDF mainly has a phase-delaying effect in the PDF receptor-expressing E-neurons of *Drosophila.* This phase-delaying effect is rather small when considering the oscillations of the molecular feedback loop (Vaze and Helfrich-Förster, 2021), but it is very large in the oscillations in resting membrane potential and firing activity (Tang et al., 2022) as well as in Ca^2+^ (Liang et al., 2016, 2017). Most interestingly, the phases of these rhythms in the s-LN_v_ (M neurons) and LN_d_ (E neurons) correlate very well with the morning and evening activity of the flies (Liang et al., 2016), confirming the role of PDF in the timing of M and E activity. PDF appears to affect two processes in the E neurons: (1) it slightly delays the molecular feedback loop via the above-mentioned mechanisms, (2) it strongly delays the rhythms in Ca^2+^, membrane potential, and action potential firing via yet unknown mechanisms. A recent study has found that the circadian Ca^2+^ oscillations depend on Ca^2+^ fluxes via the inositol trisphosphate receptor channel (ITPR), which mediates Ca^2+^ fluxes from internal endoplasmic reticulum Ca^2+^ stores (Liang et al., 2022). It is possible that PDF signaling interferes with this channel.

## 4 Communication via electrical synapses (gap junctions)

Besides communication via neuropeptides and neurotransmitters, coupling via electrical synapses plays an important role in neuronal networks, and the circadian clock network seems to be no exception (reviewed by Iyer and Sheeba, 2022). Electrical synapses are direct cell-cell connections made up of specialized structures called gap junctions. Gap junctions are clusters of intercellular channels made up of proteins called Connexins in vertebrates, Innexins in invertebrates, and Pannexins found in some chordates (reviewed in Beyer and Berthoud, 2018). Electrical synapses communicate with each other with little or no delay in transmission rate (∼0.1 ms), and electrical coupling is not limited to action potentials but also applies to subthreshold currents such as depolarization, hyperpolarization, and changes in membrane potential (Faber and Pereda, 2018).

### 4.1 The role of electrical synapses in the mammalian SCN

Although the presence of electrical synapses was demonstrated about 30 years ago in the adult SCN (Bouskila and Dudek, 1993; Colwell, 2000; Jiang et al., 1997), our understanding of how gap junctions couple circadian clock neurons and modulate circadian behavior is very preliminary. By injecting the tracer molecule Neurobiotin, Jiang et al. (1997) showed that about 30% of SCN neurons are dye-coupled and that these neurons exhibit synchronous oscillations in membrane potential. Colwell (2000) did not distinguish between neurons and glial cells and found that 73% of SCN cells showed dye coupling by using the tracer biocytin. This coupling was abolished on bath application of known gap junction blockers, strongly suggesting that these cells were indeed coupled by gap junctions. Even more interestingly, the cells showed time-of-day dependent differences in dye coupling that were stronger during daytime than during nighttime. This rhythm continued in constant darkness, indicating that it is under the control of the circadian clock (Colwell, 2000). Mouse *Connexin 36* (Cx36^−/−^) mutants showed reduced synchrony in the ultradian firing of action potentials in the SCN, and the mutant mice had weaker circadian rhythms with longer periods under constant darkness as compared to wild-type mice (Long et al., 2005). Circadian PER oscillations in the SCN of *Cx36*^*−/−*^ mutants showed a similarly lengthened period but the synchrony of PER oscillations in the different neurons was not affected, suggesting that the absence of *Cx36* lengthens the behavioral period without affecting the synchrony of circadian oscillations in individual SCN cells (Diemer et al., 2017). While *Cx36* seems to be expressed in neurons, other connexins are expressed in glial cells (astrocytes). Brancaccio et al. (2019) showed that *Cx43* plays an essential role in the communication between astrocytes and neurons in the SCN and that blocking Cx43 channels with an inhibitor interferes with the paracrine release of gliotransmitters including ATP and glutamate (see chapter 5) and slows down the period of circadian Per2-Luc oscillations in the SCN network as well as the period of the free-running behavioral rhythms.

### 4.2 The role of electrical synapses in the insect circadian master clock

Similar to mammals, gap junction blockers eliminate the synchronous firing of neurons in the aMe in cockroaches (Schneider and Stengl, 2006) and in *Drosophila*, the blocking of gap junctions in the l-LN_v_ reduces the frequency of action potential firing (Cao and Nitabach, 2008). These experiments demonstrated the presence of gap junctions in the insect master clock and their importance for the synchrony of ultradian rhythms. However, the importance of electrical synapses for circadian rhythmicity was only shown recently. Ramakrishnan and Sheeba (2021) performed a genetic knockdown screen of all eight *Drosophila* Innexin genes in the clock neurons and found that the knock-down of Innexin1 and Innexin2 lengthened the free-running period of locomotor activity. Innexin2 was present in the s-LN_v_ and l-LN_v_ and its knockdown increased the levels of PDF and the amplitude of its circadian oscillations, leading to a delay of the molecular clocks in most circadian neurons, which could ultimately explain the lengthening of the free-running period seen in behavioral rhythms (Ramakrishnan and Sheeba, 2021). While this study suggests a direct role of gap junction proteins in regulating core clock properties in a similar manner as was found in the SCN, the mechanism of how Innexin2 affects PDF levels remains unknown. A likely possibility is that subtle changes in the burst frequency of the PDF neurons affect PDF release as was shown previously (Fernandez-Chiappe et al., 2021), and that gap junctions influence these ultradian rhythms. That synchronous ultradian bursting rhythms in the clock neurons are essential for communication within the clock network was shown in the group of Donggen Luo by sophisticated dual and quadruple patch-clamp recordings of *Drosophila's* clock neurons (Tang et al., 2022). Most likely, more clock neurons are coupled with electrical synapses, and the latter contribute to their mutual coupling by promoting their synchronous ultradian firing.

## 5 Glial cells

Glial cells are among the most abundant cells in the central nervous system. They have multiple functions in the brain, ranging from guiding, supporting, and protecting neurons, to maintaining brain homeostasis, scavenging debris, cytokine signaling and immune functions, and influencing neurotransmission and synaptic connections. Likewise, glial cells are crucially involved in the circadian system and sleep regulation in mammals and insects. These functions have recently been comprehensively reviewed (Artiushin and Sehgal, 2020; Brécier et al., 2023; Carvalhas-Almeida et al., 2023; Hastings et al., 2023), and the interested reader is referred to these reviews. Here, we will only mention the most important findings, which demonstrate the close interaction of clock neurons with neighboring glial cells. Glial cells of flies and mice possess a cell-autonomous circadian clock (Brancaccio et al., 2017; Ng et al., 2011; Prolo et al., 2005; Tso et al., 2017; Zerr et al., 1990), and in the SCN the molecular clock of astrocytes is sufficient to drive circadian cycles of neuronal activity and behavior (Brancaccio et al., 2017, 2019). Similarly, the presence of PER in glial cells appears to be sufficient to generate some circadian rhythmicity in otherwise *per*^*0*^ flies (Ewer et al., 1992). Vice versa, diverse genetic manipulation of glial cells that led to dampening or loss of their circadian oscillations, simultaneously affected the behavioral rhythms of the flies (Ng et al., 2011; Ng and Jackson, 2015; Suh and Jackson, 2007; You et al., 2018). Nevertheless, the knockdown of PER in all glia did not affect the period length or degree of rhythmicity for locomotor activity in flies (Ng et al., 2011), suggesting that effects of glia on circadian behavior do not require glial clocks and are likely downstream of neuronal timekeeping mechanisms (Artiushin and Sehgal, 2020). But without doubt, glial cells communicate with the clock neurons of *Drosophila* and in this way affect their output.

Putative mechanisms of how glial cells affect clock neurons have been revealed in mammals. Astrocytes in the SCN are extensively coupled to each other by gap junctions (Welsh and Reppert, 1996), and gap junctions serve additionally the communication between glial cells and clock neurons (Brancaccio et al., 2019). In addition, glial cells can communicate with neurons via the paracrine release of neuromessengers such as ATP and glutamate, a process known as gliotransmission (reviewed by Artiushin and Sehgal, 2020). In turn, ATP and glutamate signaling are increased by the communication between glia and neurons via electric synapses as already mentioned in chapter 4.1. (Brancaccio et al., 2019). The ATP released by astrocytes is converted to adenosine within the synaptic cleft (Blutstein and Haydon, 2013). Adenosine increases homeostatic sleep but may not be involved in the coupling of circadian oscillators. ATP on the other hand might influence the SCN neurons, and indeed its extracellular level was found to oscillate with a peak during the dark phase (Womac et al., 2009). The same is true for glutamate, which shows high extracellular levels at night, coinciding with high Ca^2+^ levels in the astrocytes (Brancaccio et al., 2017). Glutamate works on inhibiting glutamate receptors on the SCN neurons and blocks their activity at night, while this inhibition is relieved during the day, thereby establishing circadian rhythms in the SCN. In summary, the communication between glial cells and clock neurons can influence not only the rhythmicity of the latter but also their mutual coupling.

A similar mechanism could also apply to the circadian clock in flies, at least as far as the LN clock neurons are concerned, which possess inhibitory glutamate receptors (Hamasaka et al., 2007) and whose neurites are located in close proximity to astrocyte-like glial cells (Helfrich-Förster, 1995).

## 6 Conclusions

There is increasing evidence that circadian timekeeping is brought about by a complex interplay between clock neurons, glial cells, and the many different factors that couple them together and enable them to function as a network. While gap junctions are thought to synchronize the ultradian firing or Ca^2+^ rhythms of different clock cells, communication via neurotransmitters, gliotransmitters, and neuropeptides seems to couple their individual circadian activity rhythms. Nevertheless, ultradian and circadian rhythms are not independent of each other (see Stengl and Schneider, 2023, for a most recent review). Therefore, the coupling of the different clock neurons is complex and works on different time scales and between different cells or parts of the clock network.

## CRediT authorship contribution statement

**Charlotte Helfrich-Förster:** Writing – original draft, Visualization, Funding acquisition, Conceptualization. **Nils Reinhard:** Writing – review & editing, Visualization.

## Declaration of competing interest

The authors declare that they have no known competing financial interests or personal relationships that could have appeared to influence the work reported in this paper.
